# “I’m pulling through because of you”: injured workers’ perspective of workplace factors supporting return to work under the Saskatchewan Workers’ Compensation Board scheme

**DOI:** 10.3389/fresc.2024.1373888

**Published:** 2024-03-15

**Authors:** Ian Lewis, Jonathan Houdmont

**Affiliations:** ^1^ Lewis Health Management Consulting Inc., Regina, SK, Canada; ^2^ School of Medicine, University of Nottingham, Nottingham, United Kingdom

**Keywords:** return to work, co-worker support, supervisor support, musculoskeletal disorders, common mental disorders, workers’ compensation

## Abstract

**Background:**

Research demonstrates sustained return to work (RTW) by individuals on medical leave is influenced by personal and job resources and job demands. Relatively few studies have been conducted in the workers’ compensation context that is known to have longer absence durations for RTW.

**Aims:**

This study sought to illuminate workers’ experience as they returned to work following a work injury that was either psychological in nature or involved more than 50 days of disability, with a focus on the co-worker, supervisor, and employer actions that supported their return.

**Methods:**

Workers in Saskatchewan, Canada, with a work-related psychological or musculoskeletal injury, subsequent disability, and who returned to work in the last three years, were invited to complete an online survey comprising of free-text questions. Thematic analysis was used to explore participants’ experiences.

**Results:**

Responses from 93 individuals were analysed. These revealed that persistent pain, emotional distress, and loss of normal abilities were present during and beyond returning to work. Almost two-thirds indicated that the supervisors’ and co-workers’ support was critical to a sustained return to work: their needs were recognized and they received autonomy and support to manage work demands. By contrast, one-third indicated that the support they expected and needed from supervisors and employers was lacking.

**Conclusions:**

Workers returning to work lacked personal resources but co-workers’ and supervisors’ support helped improve confidence in their ability to RTW. Supervisors and employers should acknowledge workers’ experiences and offer support and autonomy. Likewise, workers can expect challenges when returning to work and may benefit from cultivating supportive relationships with co-workers and supervisors.

## 1 Introduction

Supporting timely and sustained return to work (RTW) following a work-related injury is complex. Workers’ self-efficacy, RTW expectation, self-perceived work ability, and symptoms predict RTW (1, 2), while workplace factors may also facilitate or hinder the process (3). The Job Demands Resources (JD-R) theory suggests that personal resources, such as self-efficacy, have a reciprocal relationship with job resources, for example, supervisor support or modified work. Resources are motivational, buffer high job demands, supporting engagement and job performance (4). However, relatively few studies have been conducted with workers insured by a workers’ compensation scheme, resulting in a paucity of knowledge concerning factors that may support sustained RTW in this context. Therefore, this study asked injured workers in Saskatchewan, Canada, who sustained RTW to describe their experience of returning to work and workplace factors that supported the process.

Work-injury costs for workers and employers are high. Workers may experience losses of autonomy, career progression, and mental health symptoms secondary to their injury and claims experience (5). In Canada, most employers must fund a no-fault workers’ compensation insurance system that covers work-related injury wage losses and medical care and coordinates rehabilitation and RTW. The Saskatchewan Workers’ Compensation Board (SWCB) finds 75% of its claims-cost are from what it classifies as “serious injuries,” representing 13% of claims. These claims involve psychological injury or more than 50 days of partial or total disability paid. Employers with high claims costs relative to their peers can be charged up to 200% more in premiums (6). Sustainable RTW, and thus cost mitigation, is facilitated by job resources at the individual, co-worker, supervisor/line manager, employer, and overarching contextual (e.g., legislation, social system, etc.) levels (3). These are the “physical, psychological, social, or organizational aspects of the job that are functional in achieving work goals, reduce job demands, and the associated physiological and psychological costs, or stimulate personal growth, learning, and development” (7) and their presence is related to employee wellbeing (8).

Co-worker, supervisor, and employer support are influential in predicting RTW (9). A large systematic review found strong evidence that supervisor support promotes RTW for musculoskeletal disorders (MSDs) and common mental disorders (CMDs) (10). Another review of longitudinal studies for both MSDs and CMDs found co-worker combined with supervisor support predicted faster RTW. They also found some evidence that modified work and lower pre-illness job strain improved RTW outcomes (11). Similarly, a systematic review of qualitative and quantitative studies found employee-perceived care and concern, validation, communication, and trust in the organization to be related to RTW success, with invalidation of employees’ injuries or concerns related to poorer RTW outcomes (12). Reviews also found individual, co-worker, and supervisor factors to be similar for CMDs and MSDs (2, 11). A single study conducted in the workers’ compensation context found that workers with MSDs had better supervisor support and RTW than those with CMDs and the amount of support linked to RTW in both groups (13).

Qualitatively, individual studies have found that the supervisors’ positive, inclusive behaviours during RTW, procedural knowledge (14), early contact with workers off work, clearly communicated policies, modified work, and specialist case manager positions (15) are associated with improved RTW. Nielsen and Yarker (16) interviewed workers who had returned to work following a CMD and found managers could be classified as compassionate, indifferent, or demeaning with only the compassionate managers viewed favourably by workers. Meanwhile, recognition of workers returning to work from leaders and co-workers was theorized to be a foundation for expression of support behaviours and feeling supported. Specific behaviours noted to be helpful by returning workers included maintaining social contact while off work, respecting their own pace of work and limitations, providing accommodations, giving feedback, and aiding in social re-integration (17).

The literature demonstrates that RTW is driven, in part, by support experienced by workers. While much of the literature cited above may be applicable in the Canadian workplace, most studies were conducted elsewhere, involved managers and supervisors, focused primarily on white-collar employees, and were outside a workers’ compensation system. While workers’ compensation systems offer benefits to injured workers, some injured workers experience low support and helplessness (5) and lower recovery outcomes than uninsured individuals (18). This study seeks to provide insights into the experience of workers who returned to work following a work-insured injury.

## 2 Method

### 2.1 Context and sample

This study was conducted in cooperation with the SWCB. To be eligible to participate in the study a worker was required to have a “serious injury,” defined as a psychological injury or an injury with at least 50 days of partial or total disability. They were also required to have returned to work following injury. All participants had attended a rehabilitation program staffed with psychologists, physiotherapists, and exercise therapists involving 2–8 h of intervention per day, for 2–5 days per week, and successfully returned to work between 31 January 2020 and 31 January 2023. The participants’ rehabilitation and RTW were not necessarily voluntary. Like many insurers, SWCB emphasizes function over symptoms and encourages healthcare providers to make recommendations based on ability, contrary to workers who may expect to be pain-free before returning to work (19). To continue receiving benefits, participants would have been required to participate in a rehabilitation program operated by an independent rehabilitation provider. Advice on RTW would have been provided to their employer. If a participant did not agree with their rehabilitation provider's opinion of their fitness to work, the insurer would customarily defer to the provider's opinion and expect compliance.

Eligible individuals (*N *= 2,035) were identified and invited by SWCB by mail to participate. A total of 55 invitation letters were returned to sender, leaving a sampling frame of 1,980. There were 111 responses, yielding a response rate of 5.6%. Altogether 93 remained working at the time of the survey. Injury type was self-reported as “mainly physical” by 85 and “mainly psychological” by 7, with one participant not reporting. Please see Table 1 for further descriptive statistics.

**Table 1:** Participant characteristics (*n* = 93).

	*N* (%)
Working hours (after return to work from injury)	
Pre-injury hours	80 (86%)
Reduced hours compared with pre-injury	13 (14%)
Duration returned to work	
Employer had provided modified work from beginning of claim and only time off work was to attend rehabilitation	8 (9%)
Up to 3 months	7 (8%)
4–11 months	32 (34%)
1–2 years	39 (42%)
2–3 years	7 (8%)
Injury type	
Physical (strain, fracture, or other injury)	100 (90%)
Psychological (trauma, harassment, etc.)	10 (9%)
Age (at time of survey), years	
Under 30	3 (3%)
30–39	15 (14%)
40–49	20 (22%)
50–59	35 (38%)
Over 60	20 (22%)

### 2.2 Ethics approval

The study received a favourable ethical opinion from the ethics subcommittee of the Mental Health and Clinical Neuroscience Academic Unit, School of Medicine, University of Nottingham, UK. The study was conducted in accordance with the University of Nottingham's Code of Research Conduct and Research Ethics (20), which requires researchers to ensure that local regulations and customary practices are adhered to. The host organization's legal team confirmed that the study was acceptable. Moreover, the Code requires researchers to ensure that local ethical procedures and practices are reflected in the study design: this was achieved by reference to the US Department for Health and Human Services’ International Compilation of Human Research Standards—North America (21), which covers both the US and Canada. In addition, the study was conducted in accordance with the British Psychological Society Code of Human Research Ethics (22) that sets out a series of ethical principles that are compatible with those of other comparable national bodies.

### 2.3 Data collection technique

The SWCB sent eligible individuals a letter inviting their voluntary and anonymous participation. Informed consent to participate was provided by participants reading the SWCB invitation, accessing the survey website, reviewing the participant information sheet online, and clicking the link to participate. No personally identifiable information was solicited from participants. To encourage participation, individuals were invited to enter a draw for one of five $50 gift certificates to a popular online retailer, with email addresses provided on a separate survey that was not linked to survey responses.

The online survey was completed on Microsoft Forms and available from 11 April to 22 May 2023. To explore experiences of returning to work, participants were asked “What was the hardest part about returning to work?” They were also asked to describe actions performed by co-workers, supervisors, and the employer that supported their RTW. Finally, they were asked to comment on what could have been done differently to help them RTW.

### 2.4 Analysis

Inductive thematic analysis was undertaken in the method recommended by Braun and Clarke (23, 24) to explore participants’ experiences. As responses were reviewed, codes were developed, refined, and coded using NVivo software. Overall, there were 446 relevant components of response data coded. Candidate themes were developed, compared against the raw data, and further refined through reflection on the pattern and frequency of codes. Some codes were infrequent (e.g., “invalidation” reported by 9% of respondents) but important based on the literature review and warranted consideration as both semantic and latent themes were nested together in a conceptual map. Following this, a review was conducted of the physical and psychological claims separately to determine if the themes applied equally. There were only seven participants with psychological injuries and they did not generate different themes than those with physical injuries. Quotations have been edited for spelling and grammar while ensuring that the meaning remains unchanged.

### 2.5 Reflexivity

The primary researcher has practiced for two decades as an occupational therapist with work-insured individuals and as such has personal insight into the experiences of injury and RTW reported by workers, employers, and insurers.

## 3 Results

Thematic analysis generated two primary themes and three subthemes.

### 3.1 I’m not fully recovered but I’m pressing on

*I'm not fully recovered but I'm pressing on* was reported by 76% of participants and reflects their experience of low personal resources. Approximately 23% of all participants reported pain, 19% not being healed yet, 14% fear of re-injury, 13% emotional distress, 11% fatigue, and 6% were uncertain about their abilities. One person who had been back at work for 8 months described their situation as

Still having so many limitations from the injury as it is and will not be the same as it was before. It's very frustrating and hard to figure out new ways to do the things I used to do so easily [Participant (P)-31].

These participants noted that the effects of injury continued during their RTW and 24% of the participants reported continued symptoms after fully returning to work. Participant 22 reported RTW required “managing pain and being careful not to re-injure myself” and 2 years later they continued to manage pain. Another individual who had been back at work for a year after psychological injury reported “I still struggle with anxiety at work and end each day exhausted” (P-57). A few (4%) indicated their symptoms were invisible to others. Powerlessness was expressed in the latent subtheme *I'm not the same as I was but I really don't have a choice but to work.* This lack of control was evident in remarks such as “my opinion and experience didn’t really factor in when I was on my road to wellness” (P-49) and another indicated supervisors and employers should have “listened to me more … sending me back at all while I'm still at this level of pain … I'm afraid of damaging it [lower back] more returning to work so early” (P-1). The participants reported a wide range of emotions such as feeling anxious, frustrated, guilty, or abandoned as they coped with symptoms, loss of autonomy, and low confidence. Returning to work with less than full abilities created vulnerability. Participants had to request or accept help from co-workers, find new ways of working, and accept continuing discomfort.

### 3.2 You see me and care about me

This theme refers to being noticed and recognized, reflecting co-worker and supervisor behaviours that reassured participants and increased their confidence, helping sustain work performance. Almost two-thirds of the participants (65%) endorsed this theme. The supervisors received more detailed and varied comments than co-workers. Responses towards supervisors were coded as positive (36%), negative (22%), mixed (12%), or not commented on (30%).

Supervisors demonstrated regard and validation by reaching out with communication, asking how participants were doing, and modifying job demands. Their support was noted in comments such as they “expressed concern and asked how I was managing” (P-92) or “asked how my injury healed. Asked how work was going” (P-61), and appeared truly interested in participants’ wellbeing. It was noted when managers reached out to connect; for example, “my manager did regular check-ins to make sure I wasn't overdoing it” (P-53). An important subtheme was *you trust me to manage my work,* which refers to supervisors trusting workers’ selection of work activities that were helpful vs. potentially harmful, promoting autonomy. For example, “he allowed me to do the things I could still do and decline the things that I could no longer do” (P-71) or “if I needed to take an extra little break, I was free to do so. Also encouraged me to ask for help if needed” (P-83). The participants valued supervisors’ attention and connection.

Co-workers supported the participants by demonstrating concern, accepting them back to work, and helping with difficult tasks. For example, one noted that the co-workers “were very helpful in watching that I didn't do anything I wasn't comfortable with. There was no pressure or expectation from any of them, they allowed me to work at my own pace” (P-23). Another reported “I continued to work through my injury only because my co-workers were extremely accommodating” (P-53). No mention was made of supervisors encouraging co-worker support, although 33 (71%) of the 46 participants reporting co-worker support also reported supervisor support suggesting that the two could be linked.

The subtheme *you recognized me by reducing work demands* was demonstrated in reduced hours, modified schedules, and being assigned lighter or lower paced jobs. For example, “when I returned, they [co-workers] were willing to accommodate me in every way possible and never made me feel bad about it, often offering above what I needed” (P-54) and “they [my supervisor] helped me so much by slowly going back to work by giving short hours of work and less load” (P-44). Modified work required participation of the supervisor in that they “adjusted my work schedule and orders until I was back to full strength” (P-20) and comments suggested modified work was a product of supervisor and co-worker support rather than a standalone resource. Eighty-one percent who identified modified work as helpful also had codes indicating co-worker or supervisor support.

Interestingly, 47% of the participants viewed their employers as irrelevant to their RTW. Participant 53 summarized the mostly neutral response to employers:

They emailed me a few times but it was the people I work directly with who helped the most. My employer has “bigger fish to fry.” They did a check-in and delegated to my manager, which worked out good.

*You see me and care about me* was contrasted by the subtheme, expressed by 33% of participants, *but you didn't care for me when you should have.* It describes the experience of being ignored, unsupported, and not being understood while expending effort to RTW. The participants felt they were doing their part managing symptoms and wanted more from their supervisor or employer: 15% of all participants reported their supervisor or employer did not have concern for them, 11% felt low support, 11% felt the effects of their injury were not understood, and 9% felt invalidated. For example, “managers are already stressed, and having injured workers isn't helpful. I was treated with respect as far as my rights were concerned, but I never felt cared about personally. If anything I felt ignored, unimportant” (P-9). Participants expressed unmet expectations such as “disappointed, [my supervisor] never even asked how I was doing” (P-61), “I try to do my job but feel frustrated that there is no care for me” (P-84), and wanting “understanding that my injury happened at work and that I was doing my best to get back to work full time” (P-57). Some reported that employers ignored healthcare providers’ advice and “did not want to follow medical restrictions due to heavy workload” (P-45) or “tried to get me to do duties my doctor's note said I couldn't” (P-93).

## 4 Discussion

In this study, injured workers reported exerting effort to RTW amidst pain, fatigue, distress, and worry. The participants indicated that they needed to be seen and cared about, provided with assistance and modified tasks and hours, as well as trusted in their judgement on task selection and pace of work. The provision of such factors was perceived to support RTW, despite ongoing symptoms.

These findings align with earlier studies. Leader and co-worker support were identified as important, aligning with systematic reviews of longitudinal studies (10, 11). Similarly, people returning to work following CMD identified recognition of their recovery experience, modified work, respect of healthcare providers’ advice, and allowing autonomy in work duties or pace as important (17). In addition, the present study mirrors previous findings that workers find managers helpful, passive, or demeaning (16). Finally, participants’ perception of their supervisor as concerned and going out of their way to help aligns with earlier findings that the workers value empathetic and communicative managers (25). Experienced supervisors have noted that modifying work, being flexible, checking in with employees, and their own training and organizational support were helpful for RTW of their staff (26). This study also illustrated that supervisors’ and co-workers’ support helped manage pain and confidence to returning to work. Comparably, Brouwer et al.'s (27) longitudinal study of 446 Canadian work-injured clients with physical injuries found two-thirds of the variance in RTW outcome was explained by participants’ perception of RTW self-efficacy resulting from supervisor and co-worker support and ability to manage pain. Similarly, a review of 41 studies of RTW and chronic pain found managing pain, co-worker and supervisor relationships, and making workplace adjustments were deemed important by people returning to work (28).

Very few participants in the current study had experienced a psychological injury and there was no discernible pattern of results between physical or psychological claims. A review of 76 prospective studies found strong evidence that RTW self-efficacy predicted RTW for both diagnostic groups (2). In a workers’ compensation context, Smith et al. (13) found psychological claims experienced less supervisor, co-worker, and modified work support than MSD claims suggesting psychological claims needed more or different support than MSD claims.

JD-R theory (4) demonstrates that high job resources can mitigate job demands and stimulate personal resources, improving motivation and work performance. In this study, participants had low personal resources (i.e., ongoing symptoms, diminished work ability, fear of re-injury) in a context of low autonomy and daunting job demands. However, job resources of supervisor and co-worker support and reduction in job demands through modified work may have increased the personal resource of RTW self-efficacy, enhancing job performance. JD-R theory draws from the Conservation of Resources (COR) theory (29). It states those with more resources can use additional resources offered and experience a gain spiral of resources more readily than those with fewer resources. When supported adequately, participants noted feeling able, safe, and accepted, which allowed them to access job resources of modified work despite their own symptoms and other hindrances. In addition, the COR theory indicates resources often travel together, such as modified work and co-worker assistance, and are made possible by resource permissive environments or systems called resource passageways (29), and supervisors have been shown to be key resources for people returning to work (3). In this light, supportive supervisors were resource passageways because they facilitated resources such as autonomy, respect of health and abilities, and modified work. Likewise, supportive supervisors likely influenced co-worker support as the multi-level model of job resources indicates: workers are located in teams, feeding on resources from supervisors who are enabled by organizational-level resources (4).

This study is informative for injured workers and employers. Co-workers and supervisors influence RTW and supportive relationships must be cultivated by all parties. However, a power imbalance exists in the employer–worker relationship. Injured workers may reasonably expect that the employer controls the workplace and a duty of care is owed to reach out and demonstrate care for the worker. Consistent with the multi-level JD-R theory (4) and the findings of others (15, 26, 30, 31), organizations are encouraged to resource frontline leaders to develop relationships with their direct reports and take a personal interest in employees’ RTW by recognizing and validating their experience and ensuring they have the resources for success. Likewise, workers should be aware that when under strain, it may be difficult to succeed on their own and it may be helpful to ask for and accept assistance.

A strength of this study is its relatively large sample (*n* = 93) of individuals who sustained RTW and described the challenges they face. The participants were drawn from a variety of workplaces and occupations where a consistent model of care for diverse injuries was offered, thereby supporting the transferability of these findings to other workplaces. The study surveyed people who had recently returned to regular work through to those who had sustained RTW for up to 3 years. However, the study is limited in that it only included those who returned to work on a sustained basis, potentially missing opportunities to learn from people whose return was not sustained. Future studies should include such individuals to establish whether the same support issues apply. Future research could follow the lead of Aas et al. (25) and utilize in-depth interviews with returned workers, their co-workers, and supervisors to capture different perspectives on the same situation and identify barriers and facilitators for each party to promoting sustained employment of the returning worker.

While this study illustrated co-worker and supervisor behaviours that were helpful in RTW, it could not account for all the factors that may have facilitated or hindered RTW. The narrow focus of the study precluded the exploration of the barriers and facilitators in the overarching organizational environment such as healthcare providers, insurer, or unions. Moreover, data were not gathered on the contextual situation, for instance, unionization or receptiveness of the job to modification. Focusing on RTW facilitators in less permissive environments may yield insights for sectors struggling with disability prevention. Future studies could collect information on gender and obtain a larger sample to permit analyses stratified by sociodemographic characteristics. Age is related to poorer RTW outcomes (32) and older workers are representing a larger portion of workers’ compensation claims (33) and may need different forms of support than younger workers, which warrants further research. Finally, a larger sample would add strength to comparisons between psychological and non-psychological injuries.

## Data Availability

The datasets presented in this study can be found in online repositories. The names of the repository/repositories and accession number(s) can be found at https://doi.org/10.17639/nott.7371.
